# 

**Published:** 1852-04

**Authors:** 


### TO READERS AND CORRESPONDENTS.

Communications intended for publication, and all letters relating to the business of the Journal, or containing remittances in money, should be directed to Drs. Harris and Blandy, Baltimore, Md.

*Books Received.*

1. *The Half-Yearly Abstract of the Medical Sciences.* Edited by G. W. H. Rankin, M. D. Philadelphia: Lindsay & Blakiston. No. 14, July to December, 1851.

2. *Meigs' Velpeau's Midwifery.* Fourth American, with the additions from the last French edition by W. Byrd Page, M. D., &c., pp. 652. Philadelphia: Lindsay & Blakiston, 1852.

3. *The Medical Student's Vade Mecum.* By George Mendenhall, M. D. Third edition, with two hundred and twenty-four engravings. Philadelphia: Lindsay & Blakiston, 1852, pp. 690.

4. *Discourses delivered before the Cincinnati Medical Library Association.* By Daniel Drake, M. D., 1852.

5. *Review of Materia Medica for the use of Students.* By John Biddle, M. D., &c., &c. With illustrations. Philadelphia: Lindsay & Blakiston, 1852, pp. 320.

6. *The Pocket Formulary.* By Henry Beasley. First American from the last London edition: corrected, revised and enlarged. Philadelphia: Lindsay & Blakiston, 1852, pp. 443.

7. *Ten Minutes Advice respecting the Human Teeth.* By Horace Norton, M. D. Watertown, Wisconsin: Milwauke, 1851.

8. *The Importance of Preserving Teeth.* By John Linn, *Dentist.* Portsmouth, Va.

*Medical and Dental Journals Received.*

9. *The Western Medico-Chirurgical Journal*, edited by J. F. Sanford, M. D., and Samuel G. Armor, M. D., Professors in Iowa State University, January, February and March, 1852. *In Exchange.*

### *To Readers and Correspondents.*

10. *The American Journal of the Medical Sciences*, edited by Isaac Hays, M. D. Surgeon to Wills Hospital, &c., January, 1852. *In Exchange.*

11. *The New York Dental Recorder.* Edited by C. C. Allen, M. D., Dentist. January, February and March, 1852. *In Exchange.*

12. *The Dental Register of the West.* Edited by James Taylor, M. D., D. D. S. Published by order of the Mississippi Valley Association of Dental Surgeons. January, 1852. *In Exchange.*

13. *The Dental News Letter*, January, 1852. *In Exchange.*

14. *The London Medical Gazette.* January and February, 1852. *In Exchange.*

15. *Boston Medical and Surgical Journal.* Edited by J. V. C. Smith, M. D. January, February and March, 1852. *In Exchange.*

16. *The British and Foreign Medico-Chirurgical Review.* January, 1852. *In Exchange.*

17. *The Ohio Medical and Surgical Journal.* Edited by Richard L. Howard, M. D. January, 1852. *In Exchange.*

18. *The New York Journal of Medicine and the Collateral Sciences.* Edited by S. S. Purple, M. D. January and March, 1852. *In Exchange.*

19. *The Medical Examiner.* Edited by F. G. Smith, M. D., and John Biddle, M. D. January, February and March, 1852. *In Exchange.*

20. *The Southern Medical and Surgical Journal.* Edited by J. P. Garvin, M. D. January, February and March,1852. *In Exchange.*

21. *The New York Medical Gazette, and Journal of Health.* Edited by D. M. Reese, M. D., LL. D. January, February and March, 1852. *In Exchange.*

22. *The New Jersey Medical Reporter, and Transactions of the New Jersey Medical Society.* Edited by Joseph Parish, M. D. January and April, 1852. *In Exchange.*

23. *The Western Lancet and Hospital Reporter.* Edited by L. M. Lawson, M. D., and George Mendenhall, M. D. January and April, 1852. *In Exchange.*

24. *The North Western Medical and Surgical Journal.* Edited by J. Evans, M. D., and E. G. Meek, A. M., M. D. January, February and March, 1852. *In Exchange.*

25. *The St. Louis Medical and Surgical Journal.* Edited by M. L. Linton, M. D., J. S. Moore, M. D., Wm. McPheeters, M. D., and J. B. Johnson, M. D Not received.

### *To Readers and Correspondents.*

26. *The Western Journal of Medicine and Surgery.* Edited by L. P. Yandell, M. D., and T. S. Bell, M. D. Jan'y, Feb'y and March, 1852. *In Exchange.*

27. *The New Hampshire Journal of Medicine.* Edited by Edward H. Parker, A. M., M. D. January, February and March, 1852. *In Exchange.*

28. *The Charleston Medical Journal and Review.* Edited by D. J. Cain, M. D., and F. Peyrel Porcher, M. D. January and March, 1852. *In Exchange.*

29. *The Buffalo Medical Journal.* Edited by Austin Flint, M. D. January, February and March, 1852. *In Exchange.*

30. *The Provincial Medical and Surgical Journal.* Edited by W. H. Rankin, M. D., and J. H. Walsh, Esqr. January, February and March, 1852. *In Exchange.*

31. *The London Lancet,* Journal of British and Foreign, Surgical and Chemical Sciences, Chemistry, Literature and News. Editor Thomas Wakeley, M. D., for London. Sub-Editors, J. H. Bennet, M. D., and T. Wakeley, M. R. C. S. E. January, February, March and April, 1852. *In Exchange.*

32. *The Northern Lancet and Gazette of Legal Medicine.* A monthly Journal of Medical and General Science, Criticism, and Medical Jurisprudence. Edited by Horace Nelson, M. D., and Francis J. D'Avignon, M. D. January, February, March and April, 1852. *In Exchange.*

33. *The Dublin Medical Press.* January, February and March, 1852. *In Exchange.*

---

We have received the following articles which will appear in the July number.

1. Tartar of the Teeth, by J. P. Clark, Dentist, London.

2. Gratuitous Services injurious when given without due discrimination, by Dr. J. L. Levison, Brighton, England.

3. Dental Inventions and Pretensions, by R. London.

4. The Relations of Tic Douloureux to Disorders of the Teeth, by T. Dowering, M. D., London.

5. Dental Exhibitions at the World's Fair, by R. London.

6. On a New Soldering Apparatus, by Spence Bate, Dentist, Swansea, England.

7. On Amalgam Stoppings, by W. K. Bridgman, Norwich, England.

8. We have also a communication on excessive hemorrhage, after the extraction of a tooth, from London. Will the author please favor us with his name.

9. Patenting Improvements, Inventions and Discoveries in any of the branches of Medicine, by Professor W. R. Handy, M. D

---

To Delinquents.—In the July number, we intend to publish a statement of the accounts of all who have failed to pay their subscriptions, after having been *properly solicited a great number of times*

***New Subscribers.***—The rapid increase to our subscription list has exhausted Nos. 1 and 2, of vol. 2, New Series. We cannot, therefore, supply all our recent subscribers with this volume. Unless otherwise directed, we will enter their names on our books for next volume.

